# Segmented linear integral correlation Kernel ensemble reconstruction: A new method for climate reconstructions with applications to Holocene era proxies from an East Antarctic ice core

**DOI:** 10.1371/journal.pone.0318825

**Published:** 2025-04-02

**Authors:** Jason L Roberts, Lenneke M Jong, Felicity S McCormack, Anthony S Kiem, Mark A J Curran, Andrew D Moy, Jessica M A Macha, Christopher T Plummer, W John R French, Tas D van Ommen

**Affiliations:** 1 Australian Antarctic Division, Kingston, Tasmania, Australia; 2 Australian Antarctic Program Partnership, Institute for Marine and Antarctic Studies, University of Tasmania, Hobart, Tasmania, Australia; 3 Securing Antarctica’s Environmental Future, School of Earth, Atmosphere and Environment, Monash University, Clayton, Kulin Nations, Victoria, Australia; 4 Centre for Water, Climate and Land, University of Newcastle, Callaghan, New South Wales, Australia; Woods Hole Oceanographic Institution, UNITED STATES OF AMERICA

## Abstract

Understanding past climate is essential to our knowledge of how our current climate system operates, and how it might respond to future change. Techniques to reconstruct climate history are challenging, and both accuracy and certainty are hampered by the quality of the datasets used. Here we both develop a new reconstruction tool and apply it to four ice core proxy based multi-millennial Holocene climate reconstructions, chosen because of their potential influence on East Antarctic climate. The new multi-proxy reconstruction method is called Segmented Linear Integral Correlation Kernel Ensemble Reconstruction (SLICKER). This method employs a segmented linear rather than Gaussian correlation approach and builds an ensemble of reconstructions with a best fit and spread related to the best estimate of uncertainty. This method is robust for non-linear, uneven or differently sampled data and produces high-fidelity reconstructions and associated uncertainty estimates. This new method has the potential to produce more realistic reconstructions, with associated uncertainty estimates based on robust statistical measures that are insensitive to outliers. The main findings from these new reconstructions are: Antarctica temperature shows multi-decadal variability over the last twelve thousand years with increased frequency over the last two thousand years; Zonal Wave 3 index and the Southern Annular Mode both show limited trends over the last two thousand years, but an increase since the 1970s CE; and the Indian Ocean Dipole Moment index has a twentieth century CE upward trend, and a thirteenth to sixteenth century CE below average period which may be related to volcanic activity.

## Introduction

Long duration records of climate are essential for many fields of study, including detection and attribution of anthropogenic climate change (e.g. [1]), anthropology (e.g. [2]), environmental history (e.g. [3]) and palaeohydrology (e.g. [4]). Instrumental climate records are typically too short in duration to adequately characterise long-term natural variability, necessitating the reconstruction of longer-term climate records from suitable proxies. Frequently, due to the nature of the proxy archives, data may be unevenly sampled. This is especially true for ice-cores (e.g. [5]), corals (e.g. [6]), sediment cores (e.g. [7]) and tree rings (e.g. [8]). This uneven sampling potentially introduces complications when undertaking climate reconstructions, as methods to account for the uneven sampling may introduce biases. Multi-proxy reconstructions offer the potential for more robust reconstructions [9], however the uneven and different sampling of multiple proxies compound these complications.

### Reconstruction methods

While there are many methods for the reconstruction of climate fields using a spatially distributed network of proxy records, here we focus on the reconstruction of time series such as climate indices or spatially averaged climate variables reconstructed from multiple proxy records. Methods such as inverse-regression, Composite-Plus-Scale (CPS) and Principle Component Analysis have been used for some time, and have been reviewed and compared within the literature many times, for example in [10–12]. Existing multi-proxy reconstruction methods capable of handling unevenly and mismatched or differently sampled data have some shortcomings. In many methods, linear relationships are assumed between the proxies and target, or if this is not the case, then the proxy record requires transformation to enforce this linear assumption. Other proxy data transformations are often required to overcome different timescales, resolutions and missing data. However, remapping methods (e.g. [13] and [14]) may introduce errors or biases into the resulting reconstructions. CPS methods, such as used in [15], generate composites of the different proxy records by standardising the variance in all the records and then scaling the variance in the resultant reconstruction to match the variance of the target time series. The scaling method used can have a large influence on the outcome of the reconstruction. The Pairwise Comparison method [14], which compares pairs of all proxy records, improves on this by not assuming a linear relationship between proxy and target time series and also being robust to missing values. The correlation method of [16] also overcomes this issue of mismatched timescales and missing data, but the Gaussian kernel correlation that underlies this method is prone to biases towards zero [17]. Bayesian hierarchical models such as [18] are also able to incorporate uncertainties in the underlying data to improve the reconstruction.

The existing methods for palaeo-climate reconstructions discussed here each have their own strengths, weaknesses, and limitations. They perform well in many cases, but their underlying assumptions and limitations must be considered in deciding when they are an appropriate tool to use. Here we will introduce a new method capable of utilising multiple, unevenly and differently sampled proxy data-sets, for both linear and non-linear relationships, whilst producing robust reconstructions. An additional benefit of this method is that it also provides a means for assessing uncertainty over time, without the need to assume that that uncertainty is normally-distributed. There is no universally accepted best method for all situations [12] and our new method is not a panacea, but is a useful additional tool. In general, using multiple approaches and the common features of the resulting reconstructions allows for robust interpretations.

Relationships between many environmental processes are non-linear, e.g. between atmospheric pressure and wind speed (e.g. [19]), wind-speed and aerosol generation over open-water (e.g. [20]) and atmospheric water-vapour and temperature (e.g. [21]). The ability to automatically account for non-linear relationships (potentially different between all proxy and target pairs), greatly expands the utility of the new method we present here, especially when combined with intrinsic support for unevenly and differently sampled data, and provides the opportunity to produce more realistic and higher fidelity reconstructions.

### Applications

Here we present a new non-linear multi-proxy reconstruction method, Segmented Linear Integral Correlation Kernel Ensemble Reconstruction (SLICKER) which we use to produce a 12 thousand year high southern latitude annual average temperature reconstruction. This application of SLICKER demonstrates its ability to handle long reconstructions with greatly varying proxy data density and duration, whilst having an existing (lower temporal resolution) reconstruction to compare to [12].

We also present three new two thousand year climate reconstructions using SLICKER based on proxy records from an East Antarctic ice core: namely, an Indian Ocean Dipole Moment Index (DMI) reconstruction, a Zonal Wave 3 (ZW3) Index reconstruction, and a Southern Annular Mode (SAM) reconstruction. As large scale modes of climate variability, including the Southern Annular Mode and the Zonal Wave 3 are the dominant drivers of synoptic-scale atmospheric circulation governing Antarctic surface climate and sea ice changes [22], these are likely targets for Antarctic ice core based climate reconstructions for the last millennia. In addition, a dearth of multi-millennial reconstructions of these climate drivers with large high southern latitude impacts, restricts our ability to assess recent changes in a longer context: motivating our reconstruction of all three of these important indices. Additionally, our DMI reconstruction is the first to continuously cover more than 500 years, and is based on a remote proxy record (compared to the discontinuous, and local coral based proxy reconstruction of [23]). Common features between the local and remote proxy based reconstructions are more likely to represent the climate mode being investigated rather than local non-climate influences on the proxy record (such as bio-predation or nutrient limitation). Our ZW3 reconstruction is the first proxy based reconstruction, with previous reconstructions being based on climate model simulations. While there have been previous SAM reconstructions based on Antarctic ice cores proxy records, they have used linear reconstruction methods. We find that, at least for the Dome Summit South ice core proxies that we use, the proxy-SAM relationships are quite non-linear, and that non-linear methods such as SLICKER are appropriate.

Climate reconstructions using proxy records from Antarctic ice cores are a natural application of the SLICKER method due to the inherently unevenly temporally spaced data, multiple independent proxy records from individual ice cores, and non-linear proxy-climate relationships. SLICKER has intrinsic support for unevenly and differently sampled data and can automatically account for non-linear relationships between proxies and targets. This new method provides the opportunity to produce more realistic reconstructions, with associated uncertainty estimates based on robust statistical measures that are insensitive to outliers.

## Methods

Conceptually, SLICKER builds an ensemble of reconstructions in the same manner as [16]. We calculate the correlation between the target data-set and each of the proxies, and then aim to generate a reconstruction (typically of longer duration than the target) with the same correlations with each of the proxies. There are many possible reconstructions satisfying these correlations, therefore we generate an ensemble and report the robust group statistics of this ensemble.

Specifically, individual reconstruction ensemble members are initially randomly generated and then iteratively modified to minimise the mismatch in the reconstruction-to-proxy correlations compared to the corresponding target-to-proxy correlations. This procedure is repeated for all ensemble members, returning the ensemble center (central tendency from the M-estimator), uncertainty for the ensemble centre (via jack-knife resampling calibrated to yield a 95% confidence interval), and the spread of the ensemble (estimated by the Qn statistic).

The iterative modification of each ensemble member, to ensure the same correlation between the reconstruction and proxies as the target, is via a time-capped Las Vegas down gradient optimisation method with the gradients estimated via simultaneous perturbation [24]. As ensemble members are only optimized to minimise the mis-match in the correlation coefficients, both the ensemble center and spread of the reconstruction are unconstrained. Therefore, we add an offset and scale to each ensemble member to have the same ensemble center and spread as the target.

For long reconstructions with multiple proxies and large ensemble sizes, a large number of correlation calculations are required. The SLICKER FORTRAN code has been optimised to re-use calculations where possible, and utilise OpenMP shared memory parallelism. Even with these optimisations, execution time can be appreciable (from several minutes to days on modern 8 core processor computers). Therefore, we only provide FORTRAN source code, with Matlab, Python and R functionality implemented via wrappers to the FORTRAN code. For Windows users without access to a FORTRAN compiler, we also include an executable. All code and test suite examples are freely available at https://github.com/jlr581/SLICKER.

There are four key differences compared to the Gaussian kernel correlation method of [16]. First, the correlations are calculated using the Segmented Linear Integral Correlation Kernel (SLICK) method [17]. In general, this method produces less biased estimates of the Pearson correlation coefficient compared to Gaussian kernel correlation, with smaller uncertainty estimates [17]. Second, stationarity in the correlation is enhanced over the entire reconstruction period only using the subset (typically 50%) of the ensemble with the best stationarity. Third, the ensemble statistics are based on an ensemble center estimated using the M-estimator [25], while the spread is based on the Qn statistic scaled to the equivalent standard deviation for normally distributed data [26,27]. These statistical measures combine both high efficiency and robustness. Fourth, non-linearities are treated automatically (at the user’s discretion) rather than the external piecewise linear transform used in [16].

### Improved correlations

To calculate the correlations between potentially unevenly and differently sampled data, we use the Segmented Linear Integral Correlation Kernel (SLICK) method [17] to reduce error and bias compared to other methods (slotted correlation and Gaussian kernel correlation). SLICK sub-divides the computational domain into regions containing valid data points from both data-series within some threshold distance (*h*, a user selectable width parameter). Inside each region, linear interpolation is used to calculate the local integral contribution to the SLICK correlation, see [17] for details.

In cases of very uneven data spacing, individual points in the reconstruction can be decoupled from the correlation calculation. This will result in points that are unconstrained, with no meaningful information content. To reduce the occurrence of such decoupled points, we use an additional set of correlations, with a larger width parameter, which are less localised. The user can select the value of the SLICK width parameters *h*, but for most applications we recommend the default value of 0.4 [17], and 1.6 for the less localised set of correlation targets that reduce the occurrence of decoupled points. For unevenly sampled data, lower values of *h* result in more data being discarded, while larger values may result in excessive interpolation across large data gaps.

### Stationarity

Stationarity of the reconstruction can be an issue, especially for longer reconstructions. In particular, SLICKER produces an ensemble of reconstructions with prescribed correlations with the given proxies. It is possible to obtain the correct overall correlation with sub-epochs of high and low correlation, which is clearly undesirable.

During the development of the SLICKER code, several alternatives were trialled to address the issue of stationarity in the reconstruction by enforcing stationarity for each ensemble member. Attempting to enforce stationarity for each ensemble member via direct minimisation of short (50–100% of calibration epoch) and overlapping sub-window correlations during the Las Vegas down gradient optimisation was sub-optimal. It tended to produce unrealistic reconstructions with abrupt step transitions at the boundaries of the sub-windows, and more than tripled the execution time.

Instead, we have chosen not to enforce stationarity for individual members, but to select a fixed size subset of the ensemble members with better stationarity. Specifically, for each ensemble member we calculate a stationarity-index as the sum of the squared difference (over all proxies) between the reconstruction-proxy correlation for the full epoch and a running window half the length of the calibration epoch, with 50% overlap. We then select the subset of the ensemble members with the lowest stationarity-index.

### Ensemble center uncertainty calibration

To estimate the uncertainty in the ensemble center calculation, the standard deviation of a jack-knife based resample is calculated and then scaled by a calibration factor to convert this to a 95% confidence interval, based on the assumption of a normally distributed ensemble.

**Fig 1:**
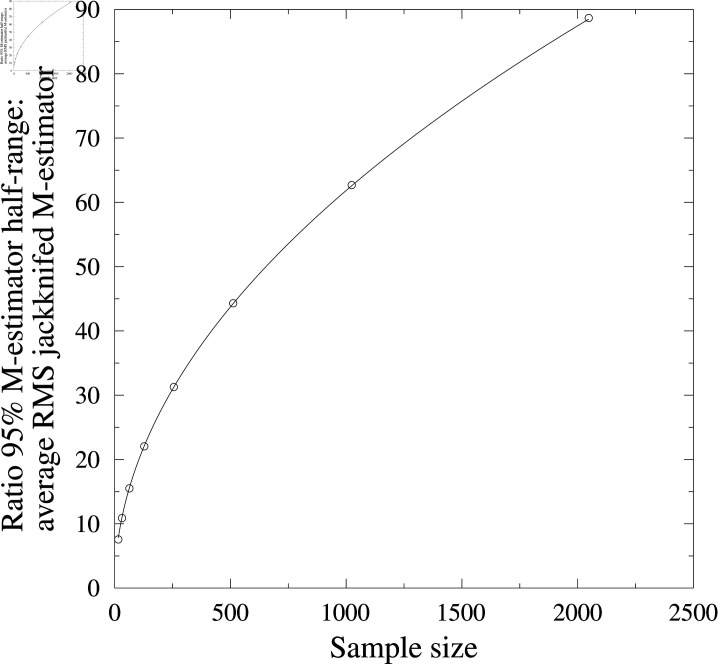
Ensemble center uncertainty calibration. To estimate the uncertainty in ensemble center calculation, the standard deviation of a resample is calculated and then scaled by a calibration factor to convert this to a 95% confidence interval, based on the assumption of a normally distributed ensemble.

The calibration factor is empirically derived by drawing *N* samples from a normal distribution with underlying mean of zero and standard deviation of one. Then the standard deviation of the resampled ensemble center is calculated. Both the ensemble center of the original sample and the resampled standard deviation are recorded. This process is repeated $2\times 10^{7}$ times and the 95% half-range of the ensemble center series is divided by the average resampled standard deviation to estimate the calibration factor for an ensemble size of *N*. This process was repeated for *N* ∈ [ 16 , 32 , 64 , 128 , 256 , 512 , 1024 , 2048 ]  (Fig 1). The resulting dataset is well fitted (in a least squares sense) by the function $cal(N)=1.956\sqrt{(}N)$.

The resampled standard deviation of the ensemble center has an inverse linear relationship with the sample size (i.e. it will halve for a doubling of the sample size). The inverse square-root calibration factor results in the 95% confidence interval reducing by a square-root factor, similar to the behaviour of traditional standard deviations with increasing sample sizes.

The underlying SLICK correlation is invariant to the addition of an offset or linear scaling to the calculated reconstruction. Therefore, similar to the Composite-Plus-Scaling method [28], we scale the reconstruction to have the same ensemble center and spread as the target data. Unlike Composite-Plus-Scaling, which uses the series mean and standard deviation for the offset and scaling respectively, we use the more robust M-Estimator and Qn. This choice results in much reduced sensitivity to outliers. However, depending on the end use of the reconstruction, other offsets and linear scalings, such as (mean, standard deviation) or (median, median-absolute-deviation) might be more appropriate. Alternative offsets and linear scalings can simply be applied as a post-processing step.

### Non-linearity

For the target series (*t*) and each proxy (*p*) with range [$p_{min}$,$p_{max}$], $\bar{R}=\frac{p_{min}+p_{max}}{2}$, $S=p_{max}-p_{min}$, we calculate the SLICK correlation for *t* with *p*, ${\left(p-\alpha \right)}^{2}$,  | *p* − *α* | × ( *p* − *α* ) , ${\left(p-\alpha \right)}^{3}$, $|p-\alpha {|}^{1∕2}$ and ${\left(p-\alpha \right)}^{1∕3}$, where $\alpha \in [\bar{R}-4S,\bar{R}+4S]$. We also (optionally) test for a stronger correlation when individual proxies have their sign inverted. We then use the target/proxy pair and corresponding *α* with the largest magnitude SLICK correlation. To simplify calculations, *α* are evenly sampled across their ranges in 800 increments. While this method formally only allows for linear, quadratic and cubic relationships between the target and proxy, for most applications this is sufficient. More complicated relationships can be allowed for by pre-processing the data.

### Test cases

Simple sinusoidal test cases from [16] are shown in S1 Appendix. These test cases include linear reconstructions in the presence of noise and missing data, non-linear and unresolved proxy components. Tables comparing SLICKER and Gaussian kernel reconstruction in terms of correlation, RMS error and reduction of error (RE) are given in S1–S3 Tables. In the majority of cases SLICKER shows equal or better correlation (14 out of 16 cases), RMS error (12 out of 16 cases) and reduction of error (14 out of 16 cases).

For more indicative test cases to show in detail, we consider three pseudo-proxy reconstructions of the 20th century annual average continental USA 2m air temperature $T_{CONUS}$ from the 20CRv3 reanalysis [29]. For the three examples, we consider IID (white), blue and red noise. The SLICKER work-flow for these test cases is given in S2 Appendix.

**Fig 2:**
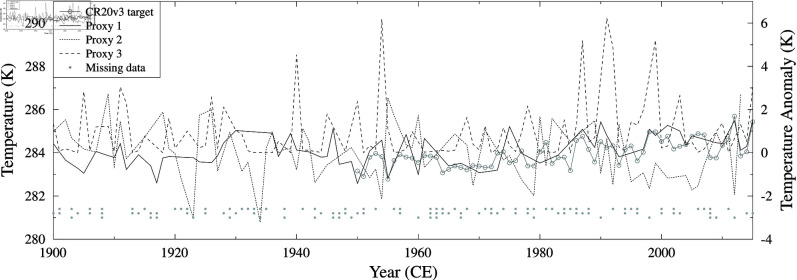
20th century annual average continental USA 2m air temperature $T_{CONUS}$ from the 20CRv3 reanalysis [29] reconstruction target (light cray line with open circles). IID noise pseudo-proxies $P_{1}$ (solid black line), $P_{2}$ (dotted black line) and $P_{3}$ (dashed black line). Location of missing data also shown at bottom of plot (gray filled circles), upper row for $P_{1}$, middle row for $P_{2}$ and lower row for $P_{3}$.

We use three pseudo-proxies ($P_{1}$, $P_{2}$ and $P_{3}$) all anomalies derived from $T_{CONUS}$ and with varying levels of missing data and added noise to reduce the correlation with $T_{CONUS}$. $P_{1}$ has 44% missing data and a Pearson correlation with $T_{CONUS}$ of 0.808. $P_{2}$ is inverted, has 38% missing data and a Pearson correlation with $T_{CONUS}$ of -0.490. $P_{3}$ has a non-linear (squared) relationship with $T_{CONUS}$, has 19% missing data and a Pearson correlation with $T_{CONUS}$ of 0.172. The reconstruction target is $T_{CONUS}$ for 1950–2015 CE and we reconstruct it for the period 1900–2015 CE. The target and IID noise pseudo-proxy datasets are shown in Fig 2.

**Fig 3:**
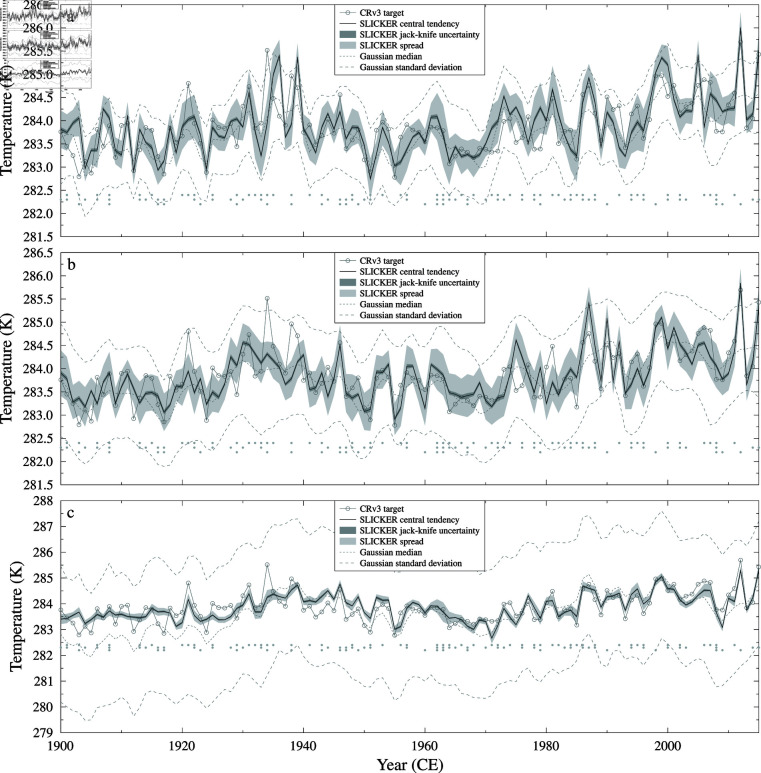
20th century annual average continental USA 2m air temperature $T_{CONUS}$ from the 20CRv3 reanalysis [29] using pseudo-proxies. SLICKER reconstruction (black line), uncertainty (dark shading) and ensemble spread (light shading) for the target (grey circles). Also shown is the median and standard deviation of the Gaussian kernel correlation reconstruction of [16] (grey dashed). a) blue noise, b) IID (white) noise, c) red noise.

Both the SLICKER and Gaussian kernel correlation reconstructed 20th century annual average continental USA 2m air temperature reconstructions are shown in Fig 3 for all three noise cases. As the added noise spectrum shifts from blue to white to red, SLICKER produces reconstructions with better correlations, lower RMS errors and more skill (higher RE). In contrast, Gaussian kernel correlation produces reconstructions with worse correlation, higher RMS error and less skill. For the blue noise case, Gaussian kernel correlation produces a marginally better reconstruction, but is noticeably worse for the IID (white) and red noise cases, see Table 1 for details.

The final test case we present is again a pseudo-proxy example, this time with an AR1 process for the target. In particular, the 200 time-sample target is given by


$$\begin{array}{ccc} AR1_{k}=0.6\times AR1_{k-1}+0.3\times \epsilon_{k} & & \end{array}$$ (1)

where *ϵ_k_* is IID noise with a mean of zero and a standard deviation of one. The two pseudo-proxies are anomalies derived from *AR*1 with added IID noise and missing data. $P_{1}$ has 35% missing data and a Pearson correlation with *AR*1 of 0.643, while $P_{2}$ has 21% missing data, a non-linear (squared) relationship with *AR*1 and a Pearson correlation with *AR*1 of 0.393.

**Fig 4:**
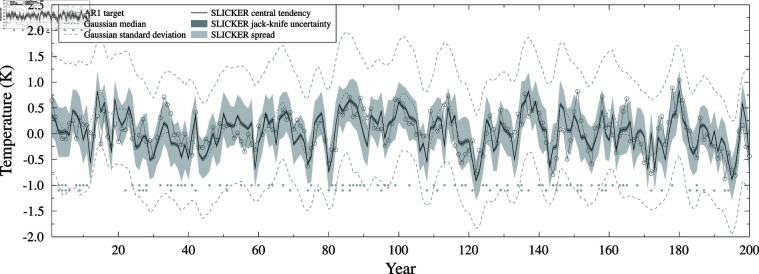
AR1 test case using pseudo-proxies. SLICKER reconstruction (black line), uncertainty (dark shading) and ensemble spread (light shading) for the target (grey circles). Also shown is the median and standard deviation of the Gaussian kernel correlation reconstruction of [16] (grey dashed).

Both SLICKER and Gaussian kernel correlation produce skillful correlations in this case (see Fig 4). Compared to the Gaussian kernel correlation reconstruction, the SLICKER reconstruction has much smaller uncertainty estimates, better correlation (0.665 compared to 0.609) and RMS errors (0.292 compared to 0.314) and is more skillful (RE of 0.345 compared to 0.243).

## Climate reconstructions

Here we present four separate climate reconstructions based on proxy records from Antarctic ice cores. Firstly, to demonstrate the utility of the SLICKER algorithm in reconstructing a climate signal from multiple proxies of differing length and variable sampling frequency, we use SLICKER to reconstruct 12 thousand years of annual average 2m air temperature averaged over 60 °S–90 °S, providing a direct comparison with the lower resolution reconstruction of [12]. We then produce three new two thousand year climate reconstructions based on proxy records from an East Antarctic ice core: namely, an Indian Ocean Dipole Moment Index reconstruction, a Zonal Wave 3 Index reconstruction, and a Southern Annular Mode reconstruction. All three reconstructions are the first multi-millennial reconstructions of their type, and in the case of the Zonal Wave 3 Index, the first proxy (rather than model) based reconstruction.

### Twelve thousand year 60 °S–90 °S temperature

For our first example, we reconstruct the 60 °S–90 °S 2m air temperature over the last 12 thousand years using proxy data from [30]. As per [12], we only consider annually resolved proxy records, and we further restrict the proxies to those that include a suitable distribution of data points and variability in the calibration period. We require proxy records with average data spacing (especially over the calibration period) to be within a factor of approximately 25 of the reconstruction data spacing to meaningfully contribute to the reconstruction. Furthermore, to allow for scaling of the reconstruction to match the target, we require sufficient variability in the proxy over the calibration period. What constitutes sufficient variability will be application specific, depending on both the reconstruction target and the other proxy records, but must a) be non-zero for each proxy record and b) be large enough to ensure the reconstruction ensemble center is not unduly quantized during the scaling step of SLICKER, resulting in a non-smooth “stair-case” reconstruction.

These two constraints, and the fact that we are reconstructing at a much higher temporal resolution than [12], result in a smaller subset of records than [12]. Specifically, we use five temperature records from the [30] dataset, derived from a combination of raw stable stable water isotope data (“PlateauRemote.MosleyThompson.1996” record from the East Antarctic Plateau [31], “TALDICE.Mezgec.2017” record from the Talos Dome ice core [32]), a linear reconstruction of temperature from stable water isotope data (“Komosomolskaia.Ciais.1992” record from multiple Antarctic ice cores [33–35]) and borehole temperature reconstructions (“LawDome.Dahl-Jensen.1999” record from the Law Dome ice core site [36] and the “WAISDivide.Cuffey.2016” record calibrated against borehole thermometry from the WAIS ice core [37]).

**Table 1:** Comparison of SLICKER and Gaussian kernel reconstruction for pseudo-proxy test case with various added noise distributions.

Noise case	Correlation	Correlation	RMS error	RMS error	RE	RE
	**(SLICKER)**	**(Gaussian)**	**(SLICKER)**	**(Gaussian)**	**(SLICKER)**	**(Gaussian)**
blue	0.723	0.721	0.443	0.428	0.426	0.465
IID (white)	0.765	0.682	0.387	0.447	0.562	0.416
red	0.790	0.647	0.362	0.632	0.619	-0.167

Unlike the centennial resolution reconstruction of [12], we reconstruct temperature every year with three different targets with approximately comparable timing (to simplify comparison): the HadCRUT 5.0.2 dataset [38] (1900–2010 CE), ModE-RA dataset [39] (1900–2008 CE) and the ERA-20C 2m air temperature for the period 1900–2010 CE [40]. While we have chosen these three reconstruction targets to highlight the influence of the target on the reconstruction, other targets such as the Last Millennium Reanalysis [41] or PHYDA [42] are equally valid targets. All three datasets were Gaussian smoothed with a three year (half power) filter to improve the performance of the reconstruction relative to the target. Of the three datasets only ERA-20C was in absolute temperature, with the other two being anomalies compared to reference epochs. As SLICKER is correlation based, we can post-hoc add a constant offset to both the HadCRUT and ModE-RA reconstructions to have the same median as the ERA-20C reconstruction to simplify comparison.

We include three reconstructions using the three different targets as there is a dearth of high southern latitude temperature data prior in the earlier part of the calibration period. Specifically, while using ERA-20C as the target for un-calibrated data is consistent with [12], it does have a post 1979 upward shift in Antarctic mean temperature [43] with increased uncertainty pre 1979 CE [44]. However, we find this shift smaller than ModE (Fig S3), possibly due to the inclusion of the fringing ocean in our analysis. ERA-20C also has higher variability than the other two target datasets.

**Fig 5:**
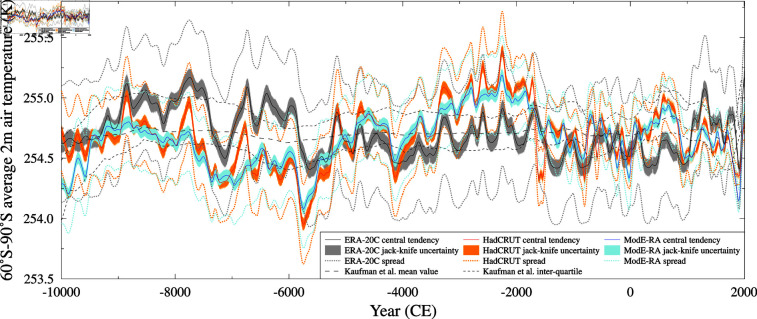
60 °S–90 °S mean temperature reconstruction. Gaussian smoothed (100 year half power) M-Estimator SLICKER reconstruction (solid lines), uncertainty (colored shading) and ensemble spread (dotted lines) for the 60°S–90°S mean temperature, 100 year (half power) for three calibration targets: ERA-20C (black), HadCRUT (red) and ModE-RA (blue). The HadCRUT and ModE-RA reconsturctions are for a temperature anomaly based target, and have had a constant offset added to have the same median value as the ERA-20C based reconstruction. Also shown is multi-method median result of [12] renormalised to have the same 1800–1900 CE mean value (long dashed line) and inter-quartiles (short dashed lines).

The three different 12 thousand year 60 °S–90 °S mean temperature reconstructions are shown in Fig 5. Their standard deviations range between 0.2–0.3 K, and the ERA-20C based reconstruction has a mean value of 254.7 K (the other two reconstructions are based on temperature anomalies, and require offsets of 254.8 K and 254.5 K for HadCRUT and ModE-RA, respectively, to have the same median value as the ERA-20C based reconstruction).

### The Dome Summit South ice core, Antarctica

Here we focus on three climate index reconstructions using proxy records from the anually resolved Dome Summit South (DSS) ice core from Law Dome, East Antarctica [45]. DSS is a very high snowfall site (0.7 metres ice equivalent y^-1^) compared to most of the Antarctic continent due to its location at the southern margin of the mid-latitudes (66.77 °S, 112.81 °E). This leads to the preservation of a unique, primarily maritime record that is highly resolved at seasonal scale for the last 2300 years [46–48]. The ice core record from DSS has been intensively studied for over three decades, resulting in a range of climate records that have been derived, including: in situ greenhouse gas analyses [49], wind [50], high latitude atmospheric pressures and moisture transport [51,52], sea ice extent [53] and annual snowfall variability [47]. More recently, regional climate proxies that exploit Law Dome’s teleconnection to lower latitudes via the synoptic to inter-annual scale variability present in the atmosphere of the southern Indian Ocean has led to proxy records of modes of climate variability such as ENSO and the IPO [54–56] and Australian rainfall and streamflow records [4,5,52,57,58]. These records have been primarily derived from the primary ice core records of seasonal sea salt aerosol concentration and annual snowfall accumulation.

Like many climate proxies, ice core based climate proxies respond to local, regional and global influences [15,59]. The remote locations of high latitude ice cores limit the local human influences [60], and so offer complementary information on remote climate processes. As such, the high resolution ice core proxy data offers the potential for annually resolved, multi-millennia reconstruction of important global climate indices. We will explore three such reconstructions, noting the possibility of future work incorporating these proxy records into multi-site reconstructions to further enhance the fidelity of the reconstructions.

In each of the three DSS proxy based climate reconstructions detailed below the skill of the reconstruction is improved by smoothing, using a 3-year (half power) Gaussian filter. In all cases the statistical significance of the reconstruction is estimated by generating 1000 synthetic target datasets using the same model used to fit the respective target. For robustness, we prefer using an ensemble center based measure of error, rather than the more typical RMS, to reduce sensitivity to outliers, and similarly a reduction of error (RE) based on the ensemble center rather than RMS values.

#### Indian Ocean Dipole Moment Index.

The Indian Ocean Dipole Moment Index (DMI) captures zonal inter-annual sea surface temperature anomalies [61]. The DMI state is known to influence east African rainfall [61] and both Australian drought [62] and bushfire risk [63]. Due to these relationships, a two thousand year reconstruction of the DMI is useful even if it is merely a manifestation of ENSO teleconnections as suggested by [64] because of its strong correlation with the NINO 3.4 index [65].

As Law Dome is highly sensitive to atmospheric pressure variability (and subsequent wind and moisture transport) across the southern Indian Ocean region, we investigated the ability of DSS proxy records to reconstruct the DMI. The propagation of signals of tropical Indian Ocean variability including the DMI to higher southern Indian Ocean latitudes is debated [66], however a number of polar studies identify signatures of the DMI, e.g. in sea ice variability [67]. A recent study of the southern Indian Ocean confirmed that certain synoptic types in the mid-latitudes of the Indian Ocean are correlated to DMI variability in austral spring, summer and autumn [68].

Our calibration target is the DMI calculated using the difference in monthly NOAA ERSSTv5 [69] sea-surface temperature anomalies between 10 °S–10 °N, 50–70 °E and 10–0 °S, 90–110 °E for the period 1854–2016 CE, then averaged annually. To assess the stationarity (or otherwise) of the DMI (units of °C), we fitted a fifth-order autoregressive (AR) model (Eq 2) to the DMI using Burg’s method [70]:


$$\begin{array}{ccc} \begin{array}{c} DMI_{K}=1.0DMI_{K-1}-0.227DMI_{K-2}\\ -0.113DMI_{K-3}-0.112DMI_{K-4}-0.143DMI_{K-5}+\epsilon_{K} \end{array} & & \end{array}$$ (2)

where the subscript *K* denotes observation number.

The order was determined by a corrected Kullback information criterion [71], and *ϵ_K_* is the unexplained variance. The (complex) roots of the associated homogeneous characteristic equation all lie within the unit circle, indicating the DMI is stationary.

To reconstruct the DMI over the last two thousand years we use proxy data from the Law Dome DSS ice core, using the stable water isotope (*δ*^18^O), winter sea-salt and summer sea-salt records [48]. As both the DMI [64] and proxy records from Law Dome [55] show relationships to ENSO, attempting to reconstruct DMI from Law Dome proxy records is reasonable. To avoid over-fitting and including proxy records that do not contribute meaningfully to the reconstruction, the selection of proxies to include was determine via generalised cross-validation [72], where proxies were only selected if their inclusion reduced the resulting rms error of the reconstruction by a sufficient amount to account for the changed degrees of freedom associated with the inclusion of the proxy.

Smoothing of both the DMI record and reconstruction with a 3 year (half-power) 1-D Gaussian filter improves the skill of the SLICKER non-linear reconstruction (ensemble center based error 0.12 °C, RMS error 0.17 °C, correlation 0.55, RE_M-est_=0.09), noting the reduction in the effective degrees of freedom, and possible reduction in statistical significance, associated with this smoothing. However, there is a downside of this smoothing, as the DMI contains a considerable amount of variability in the 1–5 year band [73], and our 3 year Gaussian filter will suppress this.

The statistical significance of the SLICKER reconstruction was estimated by using SLICK reconstructions based on the same proxy data and 1000 synthetic calibration targets generated randomly using the same AR model. Both the ensemble center error and correlation with the actual DMI reconstruction are better than 95% of the results from the 1000 synthetic AR targets.

**Fig 6:**
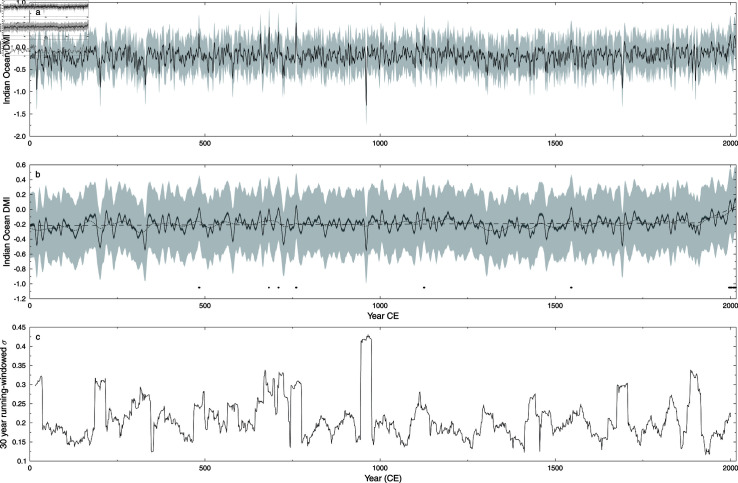
Indian Ocean Dipole Moment Index reconstruction. SLICKER reconstruction (black line), uncertainty (dark grey shading) and ensemble spread (light shading) for the Indian Ocean Dipole Moment Index. a) 3 year (half power) Gaussian smoothed M-Estimator. b) 10 year (half power) Gaussian smoothed M-Estimator, also shown 50 year (half power) Gaussian smoothed M-Estimator (dotted line), linear breakfit analysis (dashed line) showing break in linear tread at 1907 CE ± 30 years, and timing of DMI positive years (black dots) defined from 10 year Gaussian smoothed M-Estimator. c) 30-year moving windowed standard deviation of Indian Ocean Dipole Moment Index reconstruction.

The reconstructed DMI is shown in Fig 6. Note that the uncertainty of the ensemble center is almost indistinguishable from the ensemble center itself, i.e. very low uncertainty on the ensemble center calculation.

#### Zonal Wave 3 Index.

The Zonal Wave Three (ZW3) pattern is a quasi-stationary atmospheric feature of the high latitude Southern Hemisphere that captures the dominant zonal asymmetry, and is associated with meridional flow [74,75]. As such, the ZW3 is important for net poleward heat and moisture fluxes [52,76] with influences including atmospheric forcing of Antarctic climate [77] and sea ice cover [76,78,79]. Previous work [52] has shown that proxy records archived in in the DSS ice core are sensitive to the ZW3 pattern.

We reconstruct the Zonal Wave 3 (ZW3) Index of [75] using the ERA-20C [40] sea-level pressure data. The ZW3 can be calculated using either sea level pressure or 500 hPa geopotential height with strong (r ≥ 0.84) correlation between them [75].

Again, we use generalised cross-validation to select the proxies for inclusion. In this case, only the Law Dome annual mean stable water isotope was included [48]. Stationarity of the ZW3 index was assessed by fitting an AR model (Eq 3) to the time series using Burg’s method [70], with the order (third) determined by a corrected Kullback information criterion [71].


$$\begin{array}{ccc} \begin{array}{ccc} ZW3_{K} & =1.0ZW3_{K-1}-0.0909ZW3_{K-2} & \\ & -0.2366ZW3_{K-3}+\epsilon_{K} \end{array} & & \end{array}$$ (3)

As for the DMI case, the complex roots of the associated homogeneous characteristic equation all lie within the unit circle, indicating that the ZW3 is stationary.

A three year (half-power) Gaussian smoothed two thousand year reconstruction of the ZW3 index is skillful (RE_M-est_=0.02) and well correlated (*r* = 0 . 489, *p* < 0 . 05). Again, the statistical significance was estimated using SLICKER reconstructions based on the same proxy data and 1000 synthetic targets randomly generated by using the above AR(3) model. Both the ensemble center error and correlation with the actual ZW3 index are better than 95% of the results from the 1000 synthetic AR targets.

**Fig 7:**
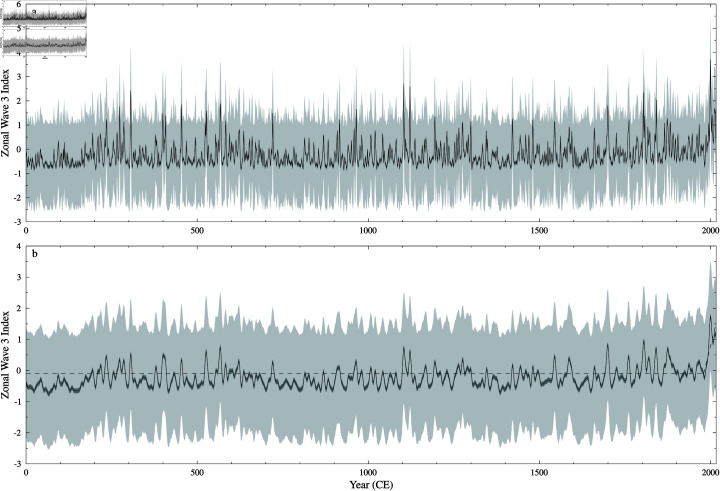
Zonal Wave 3 Index reconstruction. SLICKER reconstruction (black line), uncertainty (dark grey shading) and ensemble spread (light shading) for the Zonal Wave 3 Index. a) 3 year (half power) Gaussian smoothed M-Estimator. b) 10 year (half power) Gaussian smoothed M-Estimator, also shown linear breakfit analysis (dashed line) showing break in linear tread at 1979 CE ± 7 years.

The reconstructed ZW3 index is shown in Fig 7. The uncertainty in the ensemble center for this reconstruction, while still small, is noticeably larger than for the DMI reconstruction (Fig 6).

#### Southern Annular Mode.

The Southern Annular Mode (SAM) is the leading pattern of climate variability in the extratropical Southern Hemisphere [80,81]. The SAM describes the movement of the Southern Hemisphere westerly wind belt to the meridional migration on seasonal to centennial timescales, by characterising changes in the meridional pressure gradient across the Southern Hemisphere mid-latitudes that result from changes in the Antarctic polar front jet meridional location and intensity [81]. The SAM drives regional patterns in atmospheric circulation, temperature and precipitation from the Southern Hemisphere subtropics to the high latitudes [81]. The SAM diversely impacts Antarctic surface climate, influencing the strength of the Amundsen Sea Low [82]; near-surface temperature trends [83]; precipitation patterns [84]; Antarctic sea ice variability [85]; and Southern Ocean upwelling [86]. Due to these extensive SAM impacts on Antarctic surface climate, and the sensitivity of Law Dome to atmospheric pressure variability, we investigate the ability of the DSS proxy records to reconstruct the SAM. Such a reconstruction, extending as it does for two thousand years, provides valuable context for assessing the positive trend in summer SAM in recent decades which is currently attributed to stratospheric ozone depletion [87]. Previous SAM reconstructions, such as the [88] multi-proxy SAM reconstruction, include the DSS proxy record, but extend only over the past one thousand years, meaning the SAM reconstruction here is the first continuous record of SAM variability throughout the Common Era.

We use an extension of the [80] observation-based SAM index extending through to present (https://legacy.bas.ac.uk/met/gjma/sam.html) as our reconstruction target.

Again, we use generalised cross-validation to select the proxies for inclusion. In this case, the Law Dome annual mean stable water isotope and winter sea-salt records were included [48]. Stationarity of the SAM index was assessed by fitting an AR model (Eq 4) to the time series using Burg’s method [70], with the order (third) determined by a corrected Kullback information criterion [71].


$$\begin{array}{ccc} \begin{array}{ccc} SAM_{K} & =1.0SAM_{K-1}-0.2105SAM_{K-2} & \\ & -0.1961SAM_{K-3}+\epsilon_{K} \end{array} & & \end{array}$$ (4)

As for both the DMI and ZW3 cases, the complex roots of the associated homogeneous characteristic equation all lie within the unit circle, indicating that the SAM is stationary.

A three year (half-power) Gaussian smoothed two thousand year reconstruction of the SAM index, is skillful (RE_M-est_=0.33) and well correlated with the target (*r* = 0 . 719, *p* < 0 . 05). Again, the statistical significance was estimated using SLICKER reconstructions based on the same proxy data and 1000 synthetic targets generated by randomly using the above AR(3) model. Both the ensemble center error and correlation with the actual SAM index are better than 95% of the results from the 1000 synthetic AR targets.

**Fig 8:**
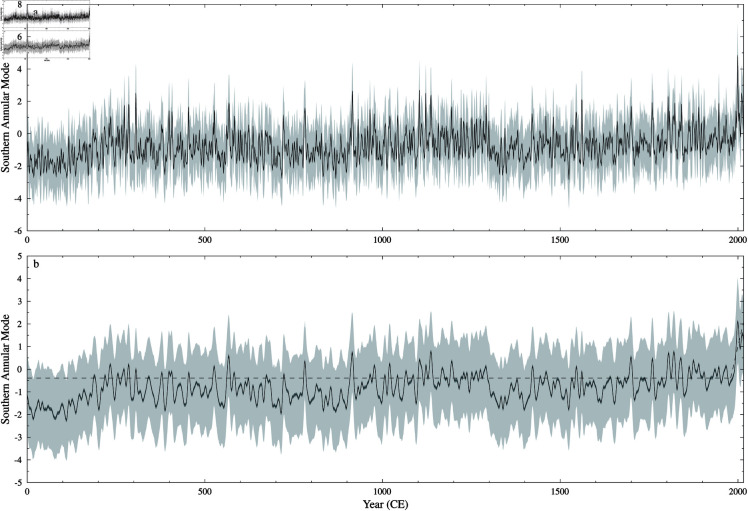
Southern Annular Mode Index reconstruction. Gaussian smoothed M-Estimator SLICKER reconstruction (black line), uncertainty (dark grey shading) and ensemble spread (light shading) for the Southern Annular Mode Index. a) 3 year (half power) Gaussian smoothed M-Estimator. b) 10 year (half power) Gaussian smoothed M-Estimator, also shown linear breakfit analysis (dashed line) showing break in linear tread at 1979 CE ± 9 years.

The reconstructed SAM index is shown in Fig 8. The two most notable features of this reconstruction are: 1) the approximate 150 year period between 0 and 150 CE that is below the long-term-average and with reduced variability in the ensemble center, 2) two extended periods below average between 785–907 CE and 1295–1418 CE.

## Discussion and conclusions

### The SLICKER method

The SLICKER method presented here is a new tool to help with the reconstruction of climate time-series (and other data series in general), with particular application to real-world data-sets. Specifically, SLICKER automatically accommodates data-sets with missing data or uneven and differently sampled data, allows for non-linear relationships between proxy and target data-series, produces robust estimates of uncertainty and calculate both uni-variate and multi-variate reconstructions. Both linear and non-linear multi-variate reconstructions allow for different relative weightings of the various proxy signals, and therefore allow for different compound curves with different trend breakpoints, even when based on the same proxy data. This is exemplified by the different reconstructions based on the same DSS ice core proxy data. In addition, all of the real-world examples present here successfully and automatically combine proxy records of different physical and chemical properties without pre-processing homogenization.

However, including multiple proxies into a reconstruction must be handled with care. In particular, checks should be undertaken to ensure that the extra degrees-of-freedom introduced by the inclusion of additional proxies, and (hopefully) reduced errors, does not come at the cost of reduced statistical significance. There are many ways to address this issue. For the DSS ice core proxy examples shown here, we have chosen to use generalised cross validation [72] to justify the inclusion of proxies. Statistical significance of the final reconstruction was checked using a statistical sampling of 1000 random draws from the fitted AR model, which is also used to check for stationarity of the target time-series. This statistical sampling from the AR model automatically accounts for the effective reduced degrees-of-freedom associated with series auto and/or cross correlations.

There are several situations where SLICKER would be particularly useful. First, meta-analysis studies that combine data from different sources which were never originally planned to be combined, and are therefore sampled at different frequencies or with a phase offset in the sampling. Second, when using proxies, such as corals and ice-cores, where uniform physical distance sampling results in irregular sampling in the time domain. Third, when one, or more, of the proxy datasets has missing data, perhaps due to instrument failure, for example rainfall datasets frequently contain missing data (e.g. [89]). Fourth, for low and variable temporal resolution proxies such as speleotherms and marine and lake sediment cores, which frequently involve non-linear transfer functions between the proxy record and the environmental parameter of interest. Fifth, when both the spread of the reconstruction and uncertainty estimates for the ensemble center are required. Unlike many methods, SLICKER inherently produces estimates of both of these quantities. Sixth, as noted in [12], there is no universal best reconstruction method, with every method having strengths, weaknesses and underlying assumptions. Using multiple different methods, with different inherent assumptions allows for robust interpretation of the reconstructions. Unlike many methods, SLICKER makes no underlying assumption about normality of datasets and uses robust statistical methods where data outliers have minimal impact on the reconstructions. However, there are some situations where SLICKER might not be the most appropriate reconstruction method. First, when there is reasonable grounds to expect a lack of long-term stationarity then SLICKER would produce erroneous results, e.g. across climate regime shifts. Second, when there is no missing data, and the relationships between the proxies and target are linear, then other computationally cheaper reconstruction methods might be more appropriate. Compared to many linear reconstruction methods, which may produce only one or a small number of reconstructions, SLICKER generates several thousand ensemble members, each of which may require tens-to-hundreds of thousand trail reconstructions. Therefore, SLICKER may take several hours to produce a reconstruction that a simpler method might produce in a few seconds.

### 12ky high southern latitude temperature

The Segmented Linear Integral Correlation Kernel Ensemble Reconstruction method was used to produce three annually resolved 12 thousand year reconstructions of average 2m air temperature between 60 °S–90 °S, each based on a different modern era target temperature time series.

Like the multi-model median reconstruction of [12], all three reconstructed temperatures are relatively constant, with no trend or significant break in slope, based on a linear break-in-slope analysis [90], although this might be impacted by the rapid temperature decrease after a reconstructed maximum temperature in 1986 CE, which might not be present if the proxy data extended further in time and allowed for a reconstruction beyond 2005 CE.

The ModE-RA based reconstruction is in good agreement with the multi-model median reconstruction of [12] with a statistically significant correlation (0.38, p < 0.05 taking into account the temperature reconstructions auto-correlations) when the ModE-RA reconstruction is sub-sampled (from the 100 year half-power Gaussian smoothed dataset) onto the same 100 year temporal resolution as [12]. Neither the HadCRUT (correlation 0.31) or ERA-20C (correlation 0.01) are significantly correlated with [12]. It should be noted that the relative constancy seen in the multi-model median reconstruction of [12] mutes the variability seen in some reconstruction methods, notably the composite plus scale. It is possible that the SLICKER method better reconstructs some of the actual Holocene variability.

The HadCRUT and ModE-RA based reconstructions are in very good agreement with each other, with a statistically significant correlation (0.80, p < 0.05 taking into account the temperature reconstructions auto-correlations), and a rms difference of 0.17 K which is around one-half the rms difference for either when compared to the ERA-20C based reconstruction, and around two-thirds of the rms difference between all three reconstructions and [12]. Neither the HadCRUT or ModE-RA based reconstructions are significantly correlated to the ERA-20C reconstruction. The differences between ERA-20C reconstruction and our other two reconstructions are small (within the standard deviation) but there is a low frequency (period of around 9950 years) small amplitude (0.3K) sinusoidal difference between them. Common features of the three temperature reconstructions are a cool period just before –7000 CE and soon after –6000 CE and a relatively warm millennium starting around –9000 CE and –3000 CE.

**Fig 9:**
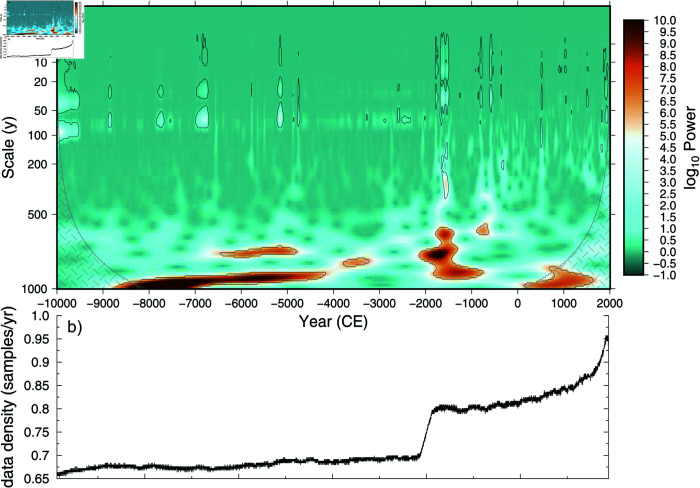
60 °S–90 °S 2m air temperature power spectrum. a) Local wavelet power spectrum of the 60 °S–90 °S mean HadCRUT temperature reconstruction using Morlet wavelets. 95% significance level for a 0.902 lag-one correlation red-noise process is shown (black lines). The cone-of-influence where edge-effects may impact the results is shown in gray hatching. b) Average combined proxy sampling density for running 256 year windows.

Local wavelet power spectrum analysis [91] of the HadCRUT based temperature reconstruction shows primarily multi-centennial variability throughout most of the 12 thousand year reconstruction (Fig 9), with some quiescent periods between -4000–0 CE. There are also intermittent periods where 5–100 year variability is significant, which appears largely independent of the proxy data sampling.

### Dome Summit South ice core based climate reconstructions

The Segmented Linear Integral Correlation Kernel Ensemble Reconstruction method was used to produce the multi-millennial Holocene climate reconstructions using proxy records from the Dome Summit South, Law Dome, East Antarctic ice core. The Indian Ocean Dipole Moment Index, Zonal Wave 3 Index and Southern Annular Mode reconstructions are all the first multi-millennial reconstructions of their type that we are aware of. Due to the non-linear relationships between these climate indices and the ice core proxies, and inherent missing data in the proxy records, SLICKER is promising reconstruction method to try. All of the reconstructions are statistically significant, skillful and include robust uncertainty estimates. All three reconstructions show no significant trend prior to the twentieth century CE, and significant upward trends thereafter, with breaks in slope around 1907 CE for the DMI and 1979 CE for both ZW3 and SAM. The start of a multi-centennial below trend epoch in the DMI reconstruction appears coincident with the 1257 CE Lombock volcanic eruption. This eruption is well resolved in the DSS ice core in other proxy records not used in our reconstructions, but with identical dating to the proxy records that were used. In both the ZW3 and SAM reconstructions the first approximately 150 years are below trend and show reduced variability. The stable water isotope proxy record appears to be the primary driver of this feature.

Each of these reconstructions are discussed in more detail below.

### DMI

The most obvious feature of the DMI reconstruction is the upward trend since the early twentieth century CE, consistent with observations of stronger warming trends in the western Indian Ocean than in the equatorial eastern Indian Ocean leading to positive DMI trend [92]. Specifically, for our reconstruction, a linear break-in-slope analysis [90], again using the more robust median and median-absolute-deviation statistics, shows a slight increasing (but not significant) trend (0.019 ± 0.012 °C millennium^−1^) prior to 1907 CE ± 30 years, followed by a significant increase in trend to 2.03 ± 0.99 °C millennium^−1^. Also noteworthy is the extended period when the DMI is below its long term average which is clearly visible on the 50 year smoothed DMI (Fig 6 dotted line) from the mid thirteenth to mid sixteenth centuries CE. The start of this downturn is coincident with the 1257 CE Lombok volcanic eruption, and extends through a period of increased volcanic activity [46]. This below trend period might be a regional reflection of the Little Ice Age, consistent with the volcanic inception posited by [93]. A decrease in Little Ice Age sea-surface temperatures in the western tropical Indian Ocean and a muted eastern Indian Ocean response [94] would lead to a decreased DMI, although there remains significant uncertainty around local timing and response magnitude. The large negative DMI value around 960 CE is only of a short duration, evidenced by the attenuation relative to other negative “spikes” in the 10 year (half power) Gaussian smoothed curve (Fig 6b), and does not coincide to a corresponding large value of any of the individual proxies. Both the uncertainty of the ensemble center and the spread of the SLICKER ensemble for this spike are consistent with the rest of the reconstruction, eliminating a temporally localised conflict between the proxies as a cause of this spike. Without additional supporting evidence of this record negative DMI event over the last two millenium, we suggest that it may be due to local processes impacting the proxy records. This highlights the caution that must be applied in interpreting reconstructions based on remote proxy data and tele-connections [23].

We are unaware of any other multi-millennial DMI reconstructions, with the longest previous reconstruction being the reconstruction of [23], a discontinuous record of six segments covering five centuries during the last 800 years. Unlike our reconstruction, [23] has monthly resolution, allowing for higher utility for climate studies. However, our longer, but lower temporal resolution reconstruction allows for some independent verification of the findings of [23]. In particular, they found a decreasing frequency of DMI positive events further back in time. Even with our lower temporal resolution, we find a similar decrease in DMI positive events (defined herein as any year where the 10 year Gaussian smoothed DMI reconstruction is positive) in the past (Fig 6b), with 18 DMI positive events after 1994 CE, but only 15 in the prior 1995 years. In terms of the variability of the DMI over the last millennium, we find broad-scale agreement with [23], with relatively low and decreasing variability in the 13th century, increasing variability in the 16th century, high variability in the latter part of the 17th century and relatively low but increasing variability in the latter part of the 18th century (Fig 6c). Contrary to [23], we find enhanced variability in the late 15th century and between approximately 1850–1930 CE, although neither epoch is associated with DMI positive events.

### ZW3

This is the only known ZW3 reconstruction from proxy data, although ZW3 derivations from model simulations have been reported through the deglacial period [95], over the past 500 years [96], through the twentieth century CE [76] and projected through the twenty first century CE [79].

The proxy reconstruction here shows no overall trend in ZW3, as also generally seen in the simulations through both the pre-twentieth century CE and late twentieth century CE. The ZW3 reconstruction does have two notable features: 1) the period of approximately 150 years between 0 and 150 CE that is below the long-term-average and exhibits reduced variability, and 2) the upward trend from the late twentieth century CE. Linear break-in-slope analysis [90] shows a statistically significant break at 1979 CE ( ± 7 years) with a trend afterwards of 0.044 ± 0.013 year^−1^. This is broadly consistent with the findings of [76], who found an increasing trend in the period 1960–2005 CE of 0.386 over the 45 years due to a more meridional pattern of circulation associated with global warming. This trend is approximately one-fifth the trend we find, consistent with their Index being based on 500 hPa geopotential heights, and therefore being more attenuated than our sea-level pressure based Index. [76] also note the timing of the shift occurring in the late 1970s CE, pointing to other large scale atmospheric changes around this time. Onset of long-term drought in South-West Western Australia (SWWA) has been characterised by an change in winter rainfall in 1971 CE ( ± 7 years) [97]. This drought has been linked via ZW3 influence to meridional flow and increased snowfall at Law Dome [52].

The late twentieth century CE shift is not generally seen in twentieth century CE simulations [76], suggesting that internal, rather than forced, variability may be the cause. Model simulations (1900–2100 CE, historical and SSP5–8.5) show no ZW3 trend in the multi-model mean [79] despite climatic warming through this period. Turning to a period of very different boundary conditions, simulation of Antarctic warming during Heinrich Stadial 1 does show strengthening ZW3 trend that contributed to Antarctic warming [95].

While there are no other known proxy based ZW3 reconstructions, long term paleo-climate reanalysis including sea-level pressure allow for the estimation of a historical ZW3. Unlike our reconstruction, both of the long-term paleo-climate reanalysis based ZW3 indices show a pre-industrial downward trend. Specifically, the ZW3 index based on 1421–2008 CE reconstruction [39] shows a downward trend (-0.069 ± 0.036 century^−1^) prior to 1905 CE ( ± 18 years) followed by a significant upward trend (1.261 ± 0.332 century^−1^). The common era reanalysis [41] also shows a significant early downward trend (-0.066 ± 0 . 021century) in this case prior to 1682 CE ( ± 145 years) followed by a upward trend (0.271 ± 0.256 century^−1^). However, the ZW3 index, being based on the location of three Southern Hemisphere atmospheric pressure centres, is challenging to reconstruct, with neither of these two reanalysis based ZW3 (or a ZW3 calculated using observationally based sea-level pressure data [98]) agreeing, even over the (relatively) observationally rich 20th century (see S4 Fig). This highlights the need for additional multi-millennial ZW3 reconstuctions to allow for the identification of robust common features.

Notwithstanding the model results, it is clear from this long proxy reconstruction that the change since the 1970s is unusual – there is no comparable positive anomaly in two millennia. This sharpens focus on whether internal variability may indeed play a role. However, offset against this is the sustained period of low ZW3 in the first 150 years of the reconstruction which has no obvious forcing and would appear to be an example of internal variability at least causing a decrease in zonal asymmetry.

### SAM

Like the DMI and ZW3 reconstructions, the two thousand year Southern Annular Mode reconstruction shows a upward trend from the late twentieth century CE. Specifically, a linear break-in-slope analysis [90], shows no trend (slope 0.000 ± 0.000 year^−1^) prior to 1979 CE ± 9 years, followed by a statistically significant break in slope of 0.068 ± 0.025 year^−1^.

Compared to the one thousand year SAM reconstructions of [88] and [99], the new two thousand year SAM reconstruction is more attenuated, and trendless: a similar linear break-in-slope analysis applied to the one thousand year SAM reconstruction of [88] shows statistically significant slopes (–0.003  ± 0.001 and 0.006 ± 0.001 year^−1^ respectively before and after 1520 CE ± 59 years), and when applied to the one thousand year annual (correlation plus stationarity proxy selection) SAM reconstruction of [99] also shows statistically significant slopes (–0.001 ± 0.000 and 0.003 ± 0.001 year^−1^ respectively before and after 1663 CE ± 57 years). All three reconstructions share many common features, such as a relatively quiescent, but slightly above average, period from around 1100 to 1300 CE, a below average century starting around 1400 CE, and slightly elevated values post 1800 CE. However, our reconstruction noticeably differs from the other two between 1300 to 1400 CE with a prolonged below average epoch, while the two other reconstructions have some of their highest values. This discrepancy may be due to the lack of perfect SAM zonal symmetry [100] combined with the various proxy records sampling different regions of the globe.

The SLICKER method presented here is a new approach to reconstructions of climate proxies. Although it comes at a greater computational expense than some other methods, it enables flexibility where data are unevenly sampled and for climate processes that are inherently non-linear, and allows a robust estimate of uncertainty. We have applied the SLICKER method to proxy records from Antarctic ice cores, generating a new reconstruction of 12ky temperature, and, for the first time, reconstructions of the DMI, ZW3 and SAM that extend over the last two thousand years. These reconstructions have significant potential utility in characterising pre-industrial climate variability, over larger spatial domains than may be represented by current reconstructions.

## Supporting information

S1 Appendix Test cases.
(PDF)

S2 Appendix Pseudo-proxy test case work-flow.
(PDF)

S3 Fig **60 °S–90 °S temperature targets.** High latitude southern hemisphere temperature targets for the 12 thousand year temperature reconstruction. ERA-20C (solid line) [40] Modern Era Reanalysis (ModE-RA) (dashed line) [39] and HadCRUT 5.0.2 (dotted line) [38].
(PDF)

S4 Fig **ZW3 reconstructions from paleo-climate reanalysis.** ZW3 indices calculated from three centennial to multi-millennial paleo-climate reconstructions, Modern Era Reanalysis (ModE-RA) (solid line) [39], Hadley Centre Sea Level Pressure dataset (HadSLP2) (dotted line) [98] and Last Millennium Reanalysis (LMRv2) (dashed line) [41].
(PDF)
