# Supplementary material for: Segmented linear integral correlation Kernel ensemble reconstruction: A new method for climate reconstructions with applications to Holocene era proxies from an East Antarctic ice core
Source: PLoS One. 2025 Apr 2;20(4):e0318825. doi: 10.1371/journal.pone.0318825 (PMC11964464; doi:10.1371/journal.pone.0318825)
Supplement: S1 Appendix — Test cases. (PDF) [file pone.0318825.s001.pdf]

Segmented Linear Integral Correlation Kernel Ensemble Reconstruction: A new method for climate reconstructions with applications to Holocene era proxies from an East Antarctic ice core

Jason L. Roberts[1,2*], Lenneke M. Jong[1,2], Felicity S. McCormack[3], Anthony S. Kiem[4], Mark A.J. Curran[1,2], Andrew D. Moy[1,2], Jessica M.A. Macha[3], Christopher T. Plummer[1,2], W. John R. French[1,2] and Tas D. van Ommen[2]

1 Australian Antarctic Division, Kingston, TAS 7050, Australia

2 Australian Antarctic Program Partnership, Institute for Marine and Antarctic Studies, University of Tasmania, Hobart, TAS 7004, Australia

3 Securing Antarctica's Environmental Future, School of Earth, Atmosphere & Environment, Monash University, Clayton, Kulin Nations, Victoria 3800, Australia

4 Centre for Water, Climate & Land, University of Newcastle, Callaghan, New South Wales 2308, Australia

* Jason.Roberts@aad.gov.au

Supporting information

S1 Appendix Test cases. We consider the test cases from [1], namely linear reconstructions in the presence of noise and missing data, non-linear and unresolved proxy components.

Noise The first test case is for the reconstruction of a two component sinusoidal signal with additive independent and identically distributed (IID) noise (ν). Specifically, the target (t) is given by $t = \sin(x/10) + 0.4\sin(x/3)$ and the two proxies are $\sin(x/10)$ and $\sin(x/3)$. S1 Fig shows the reconstructions for five different noise levels. For this evenly sampled case, both methods are essentially indistinguishable and the median

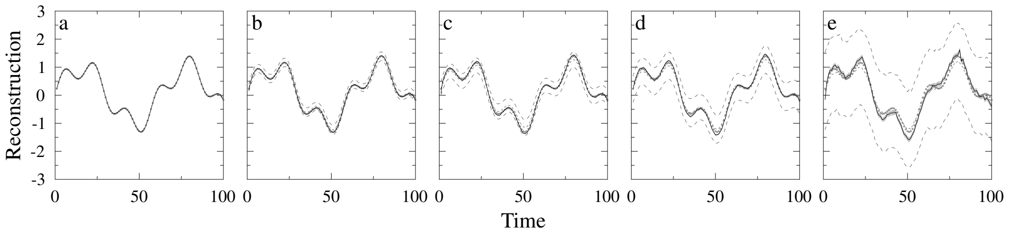

Fig 1. SLICKER noise test cases. Ensemble center SLICKER reconstruction (black line) and 95% confidence interval (shading) for the target (grey circles) $t = \sin(x/10) + 0.4\sin(x/3) + \theta\nu$, where ν is IID noise and a) $\theta = 0$, b) $\theta = 0.1$, c) $\theta = 0.2$, d) $\theta = 0.4$ and e) $\theta = 1.0$. Also shown is the median and standard deviation of the Gaussian kernel correlation reconstruction of [1] (grey dashed).

reconstruction shows good fidelity, with slight under-prediction of local extrema with increasing noise levels, see Table S1 for statistics for the two methods.

Table S1. Comparison of SLICKER and Gaussian kernel reconstruction for noise test cases, $t = \sin(x/10) + 0.4\sin(x/3) + \theta\mu$, where μ is IID noise.

Noise level	Correlation (SLICKER)	Correlation (Gaussian)	RMS error (SLICKER)	RMS error (Gaussian)	RE (SLICKER)	RE (Gaussian)
$\theta = 0$	1.000	1.000	0.001	0.000	1.000	1.000
$\theta = 0.1$	1.000	1.000	0.016	0.043	1.000	0.996
$\theta = 0.2$	1.000	1.000	0.037	0.082	0.997	0.987
$\theta = 0.4$	1.000	1.000	0.051	0.070	0.995	0.990
$\theta = 1.0$	0.992	0.997	0.159	0.096	0.950	0.982

Missing data The second test case is for the reconstruction of a two component sinusoidal signal with various rates of missing data. Specifically, the target (t) is given by $t = \sin(x/10) + 0.4\sin(x/3)$ and the two proxies are $\sin(x/10)$ and $\sin(x/3)$ with up to 67% of each proxies data missing. S2 Fig shows the reconstructions for four different noise levels. For all missing data rates, both methods median reconstructions show good fidelity, with SLICKER producing better correlations and smaller uncertainty estimates at higher missing data rates, see Table S2 for statistics for the two methods.

Table S2. Comparison of SLICKER and Gaussian kernel reconstruction for missing data test cases.

% missing data proxy1/proxy2 (noise)	Correlation (SLICKER)	Correlation (Gaussian)	RMS error (SLICKER)	RMS error (Gaussian)	RE (SLICKER)	RE (Gaussian)
0/0	1.000	1.000	0.001	0.000	1.000	1.000
67/0	1.000	1.000	0.003	0.012	1.000	1.000
20/20	1.000	0.990	0.005	0.110	1.000	0.976
40/40	0.996	0.988	0.082	0.119	0.987	0.972
20/20($\theta = 0.4$)	0.994	0.987	0.093	0.119	0.983	0.972

Non-linear The final test case is for unresolved proxy components and non-linear

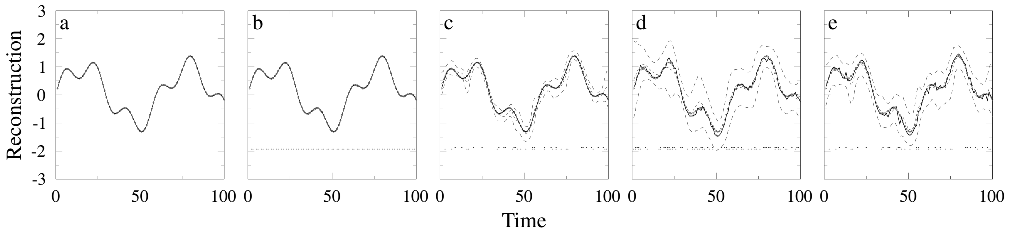

Fig 2. SLICKER missing data test cases. Ensemble center SLICKER reconstruction (black line) and 95% confidence interval (shading) for the target (grey circles) $t = \sin(x/10) + 0.4\sin(x/3) + \theta\nu$, where ν is IID noise ($\theta = 0$ for a–d and $\theta = 0.4$ for e). Location of missing data shown at bottom of plot (light grey for $\sin(x/10)$, dark grey for $\sin(x/3)$). a) no missing data b) 67% missing for $\sin(x/10)$ c) 20% missing from both proxies, d) 40% missing from both proxies (note using non-default SLICK width parameter - see discussion) and e) 20% missing from both proxies and $\theta = 0.4$ noise. Also shown is the median and standard deviation of the Gaussian kernel correlation reconstruction of [1] (grey dashed).

reconstructions of a two component sinusoidal signal. Specifically, a low-frequency unresolved proxy of $\sin(x/20)$ and the non-linear targets of $t = \sin(x/10) + 0.4\sin^2(x/3)$ and $t = \sin^2(x/10) + 0.4\sin(x/3)$ are shown in S3 Fig. As expected, the failure to resolve all the proxies reduces the fidelity of the median reconstruction and increases the uncertainty for both methods, although SLICKER has slightly reduced uncertainties. For non-linear proxies, the uncertainties for SLICK are substantially smaller than for Gaussian kernel correlation, see Table S3 for statistics for the two methods.

Table S3. Comparison of SLICKER and Gaussian kernel reconstruction for non-linear test cases. a) unresolved proxy component of $\sin(x/20)$, b) non-linear target of $\sin(x/10) + 0.4\sin^2(x/3)$ and c) non-linear target of $\sin^2(x/10) + 0.4\sin(x/3)$.

Test case	Correlation (SLICKER)	Correlation (Gaussian)	RMS error (SLICKER)	RMS error (Gaussian)	RE (SLICKER)	RE (Gaussian)
a linear	0.610	0.588	0.721	0.746	0.282	0.231
b linear	0.978	0.978	0.159	0.168	0.945	0.939
c linear	0.650	0.617	0.377	0.359	0.274	0.341
a non-linear	0.636	0.639	0.706	0.740	0.312	0.244
b non-linear	0.999	0.996	0.032	0.082	0.998	0.986
c non-linear	1.000	0.984	0.002	0.089	1.000	0.960

References

1. Roberts JL, Tozer CR, Ho M, Kiem AS, Vance TR, Jong LM, et al. Reconciling Unevenly Sampled Paleoclimate Proxies: A Gaussian Kernel Correlation

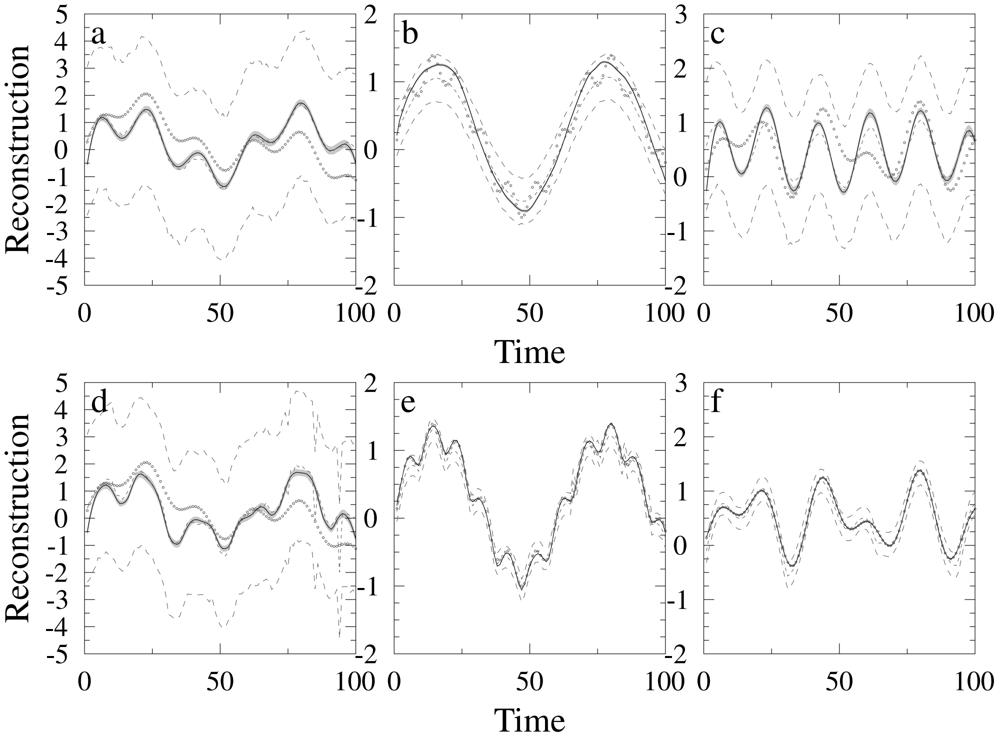

Fig 3. SLICKER non-linear test cases. Ensemble center SLICKER reconstruction (black line) and 95% confidence interval (shading) for the target (grey circles) for linear (top row) and non-linear (bottom row) reconstructions for $t = \sin(x/10) + 0.4\sin(x/3)$. a) and d) unresolved proxy component of $\sin(x/20)$, b) and e) non-linear target of $t = \sin(x/10) + 0.4\sin^2(x/3)$, c) and f) non-linear target of $t = \sin^2(x/10) + 0.4\sin(x/3)$. Also shown is the median and standard deviation of the Gaussian kernel correlation reconstruction of [1] (grey dashed).

Multiproxy Reconstruction. Journal of Environmental Informatics. 2020;35(2):118–127. doi:10.3808/jei.201900420.
