# Supplementary material for: Segmented linear integral correlation Kernel ensemble reconstruction: A new method for climate reconstructions with applications to Holocene era proxies from an East Antarctic ice core
Source: PLoS One. 2025 Apr 2;20(4):e0318825. doi: 10.1371/journal.pone.0318825 (PMC11964464; doi:10.1371/journal.pone.0318825)
Supplement: S2 Appendix — Pseudo-proxy test case work-flow. (PDF) [file pone.0318825.s002.pdf]

# Segmented Linear Integral Correlation Kernel Ensemble Reconstruction: A new method for climate reconstructions with applications to Holocene era proxies from an East Antarctic ice core

Jason L. Roberts[1,2*], Lenneke M. Jong[1,2], Felicity S. McCormack[3], Anthony S. Kiem[4], Mark A.J. Curran[1,2], Andrew D. Moy[1,2], Jessica M.A. Macha[3], Christopher T. Plummer[1,2], W. John R. French[1,2] and Tas D. van Ommen[2]

**1** Australian Antarctic Division, Kingston, TAS 7050, Australia

**2** Australian Antarctic Program Partnership, Institute for Marine and Antarctic Studies, University of Tasmania, Hobart, TAS 7004, Australia

**3** Securing Antarctica's Environmental Future, School of Earth, Atmosphere & Environment, Monash University, Clayton, Kulin Nations, Victoria 3800, Australia

**4** Centre for Water, Climate & Land, University of Newcastle, Callaghan, New South Wales 2308, Australia

* Jason.Roberts@aad.gov.au

## Supporting information

**S2 Appendix   Pseudo-proxy test case work-flow.** The internal work-flow for SLICKER is given below using default values for SLICK width parameters ($h$ of 0.4 and 1.6) and the best 10% of ensemble members from a stationarity point-of-view.

```
1. Calculate SLICK correlations between target and proxies
 1a) using h=0.4 to get correlations of 0.801, -0.505 and 0.270
     for P1, P2 and P3 respectively
 1b) using h=1.6 to get correlations of 0.773, -0.380 and 0.297
     for P1, P2 and P3 respectively
```

```
 1c) check for best non-linear correlation using h=0.4, to get
     non-linear correlations of -0.812, -0.534 and -0.345
     for P1, P2 and P3 respectively
2. Foreach member in ensemble
 2a) guess initial test reconstruction
 2b) calculate correlations for h=0.4, h=1.6 and non-linear h=0.4
 2c) calculate the rms difference between the correlations in step
     1 and 2b.  If below threshold skip steps 2d-2h
 2c) loop until converged
   2d) if step 2c has taken longer than allowed, start again at step 2a
       with a new initial guess
   2e) modify test reconstruction via simultanous pertubation
   2f) calculate correlations for h=0.4, h=1.6 and non-linear h=0.4
   2g) calculate the rms difference between the correlations in step
       1 and 2f.  If rms difference is improved keep modification,
       otherwise discard modification
   2h) if the rms difference from step 2g is below threshold exit step 2c
3. Now have an ensemble of 4096 reconstructions each with a rms difference
   between the correlations in steps 1 and 2b/2g below the required
   threshold
 3a) Calculate the stationarity index for each ensemble member which is the
     squared difference between correlations over the full epoch and
     correlations for sub-windows of the reconstruction.
 3b) Select the 10\% of ensemble members (409 in total) with the best
     stationarity index
4. Calculate ensemble statistics (ensemble center, uncertainty and
     spread) on the 409 member subset.
```
