# Supplementary figures and images for: Segmented linear integral correlation Kernel ensemble reconstruction: A new method for climate reconstructions with applications to Holocene era proxies from an East Antarctic ice core
Source: PLoS One. 2025 Apr 2;20(4):e0318825. doi: 10.1371/journal.pone.0318825 (PMC11964464; doi:10.1371/journal.pone.0318825)

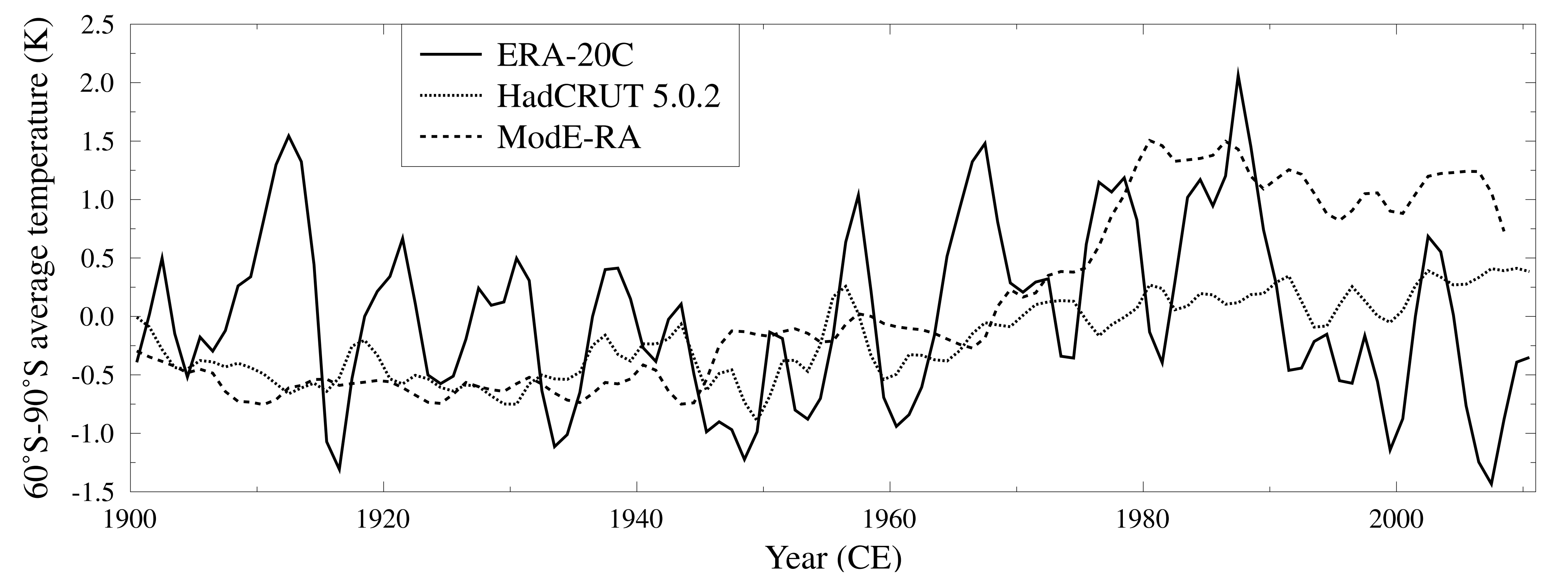

Supplement: S3 Fig — 60 °S–90 °S temperature targets. High latitude southern hemisphere temperature targets for the 12 thousand year temperature reconstruction. ERA-20C (solid line) [40] Modern Era Reanalysis (ModE-RA) (dashed line) [39] and HadCRUT 5.0.2 (dotted line) [38]. (PDF) [file pone.0318825.s003.pdf]

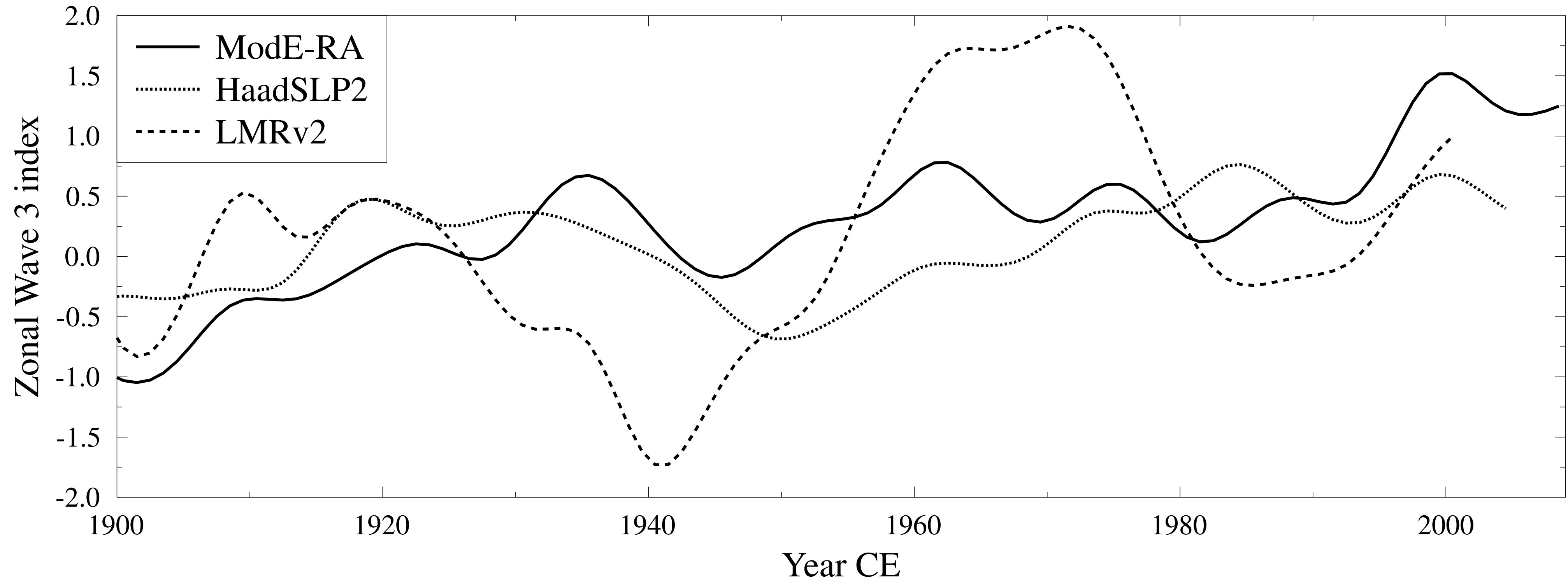

Supplement: S4 Fig — ZW3 reconstructions from paleo-climate reanalysis. ZW3 indices calculated from three centennial to multi-millennial paleo-climate reconstructions, Modern Era Reanalysis (ModE-RA) (solid line) [39], Hadley Centre Sea Level Pressure dataset (HadSLP2) (dotted line) [98] and Last Millennium Reanalysis (LMRv2) (dashed line) [41]. (PDF) [file pone.0318825.s004.pdf]
